# Usefulness of Capillary Gel Electrophoresis-Based PCR for Detection of *Clostridioides difficile* Strains with Hypervirulent Ribotypes

**DOI:** 10.3390/gels10050343

**Published:** 2024-05-17

**Authors:** Tomasz Bogiel, Alicja Dura, Marcin Woźniak, Agnieszka Mikucka, Piotr Kanarek

**Affiliations:** 1 Department of Microbiology, Ludwik Rydygier Collegium Medicum in Bydgoszcz, Nicolaus Copernicus University in Toruń, 85-094 Bydgoszcz, Poland; a.mikucka@cm.umk.pl; 2 Department of Clinical Microbiology, Antoni Jurasz University Hospital No. 1, 85-094 Bydgoszcz, Poland; 3 Department of Forensic Medicine, Ludwik Rydygier Collegium Medicum in Bydgoszcz, Nicolaus Copernicus University, 87-100 Toruń, Poland; alicja_mulyk@wp.pl (A.D.);; 4 Department of Microbiology and Food Technology, Faculty of Agriculture and Biotechnology, Bydgoszcz University of Science and Technology, 85-029 Bydgoszcz, Poland

**Keywords:** 027 ribotype, capillary gel electrophoresis, *Clostridioides difficile*, hypervirulence, ribotyping, Webribo database

## Abstract

*Clostridioides difficile* is a complex of anaerobic bacteria responsible for the epidemics of post-antibiotic diarrhea as one of the examples of CDI (*Clostridioides difficile* infection). As many as 70% of cases concern hospitalized patients, particularly those in intensive care units. Ribotyping is one of the most common methods for differentiating bacterial strains. The purpose of this work was to show the effectiveness of the gel electrophoresis-based PCR ribotyping method and the Webribo database for typing *C. difficile* isolates, including the hypervirulent 027 ribotype. DNA samples extracted from 69 *C. difficile* strains with previously marked genotypes were included in this study. PCR was performed using 16S–23S primers, and capillary gel electrophoresis was performed on the Applied Biosystem 3130xl Genetic Analyzer. The Webribo database was applied for ribotype assignment. Out of 69 samples, 48 belonged to already known ribotypes, 13 represented new ribotypes and 8 was indicated as similar to the existing ones, having some differences. Capillary gel electrophoresis-based PCR is an effective method for the differentiation of *C. difficile* ribotypes and can be recognized as a very useful tool in epidemiological studies, while the Webribo database is a useful and an accessible database for a quick analysis of *C. difficile* ribotypes.

## 1. Introduction

Capillary gel electrophoresis is a molecular technique of amplified DNA separation that provides advantages in terms of analytical simplicity, high separation efficiency, low sample and solvent volume consumption. The short time necessary for data analysis and its applicability to a wide range of substances of biomedical importance makes it increasingly more popular in different kinds of analytical laboratories, including molecular biology and microbiological labs [1]. It has also been proven that applying appropriate sequences (e.g., 16S-23S, ITS) in capillary gel electrophoresis is useful for bacteria and yeast identification [2,3]. Therefore, capillary gel electrophoresis has already been applied to a number of research studies dedicated to particular microbial investigation and differentiation, including viruses [4,5], bacteria [6,7,8,9,10] and fungi [11]. Other application aspects are especially dedicated to foodborne pathogen detection [8,12,13,14,15] and their typing [16,17,18,19,20].

*Clostridioides difficile* bacteria are a Gram-positive, toxin- and spore-producing obligate anaerobes with a broad spectrum of distribution in the environment [21]. The microorganism was first isolated in the 1930s from the feces of healthy infants and has since been classified as a part of the natural human microbiota. The association of *C. difficile* with post-antibiotic diseases was not discovered until the 1970s [21].

This bacterium is the causative agent of healthcare-associated diarrhea. It typically arises from prolonged therapy with clindamycin, third- and fourth-generation cephalosporins, fluoroquinolones, and proton pump inhibitors [21,22]. Additional factors contributing to the development of the disease include advanced age, compromised immunity, and hospitalization [23,24]. It is important to note that *C. difficile* infections (CDI) are gaining increased attention from clinicians around the world due to the continued rise in the number of cases of these infections [24,25]. High morbidity and mortality rates make CDI considered one of the most important infections in North America and Europe [25,26,27,28].

The prevalent diagnostic criteria typically involve diarrhea, manifesting at least five times daily, along with symptoms indicative of toxic megacolon (toxic colonic dilatation syndrome). Additional indicators encompass abdominal pain, elevated fever, escalating leukocytosis, clinical signs of peritonitis and an overall deterioration of health [23,28].

The disease’s progression is linked to an imbalance in the intestinal microbiota’s homeostasis due to prolonged antibiotic exposure. The pathogenesis involves an elimination of certain competing bacteria which reduce environmental pressure, facilitating an unrestricted proliferation of *C. difficile* [23,28]. An excessive pathogen proliferation is further promoted by virulence factors: toxin A (tcdA) and toxin B (tcdB), encoded at the pathogenicity locus (PaLoc), which inactivate Rho and/or Ras GTPases by glucosylation [29,30]. Moreover, during the early 2000s, the emergence of a hypervirulent, epidemic ribotype 027 (027/NAP1/BI) was noted. This strain not only demonstrated higher resistance to fluoroquinolones but also, due to some mutation, showed an increased expression of the pathogenicity locus and the capability to produce another toxin: binary *C. difficile* toxin (*C. difficile* transferase-CDT) [30,31,32]. Since its discovery, ribotype 027/NAP1/BI has been regarded as the most significant hypervirulent ribotype of *C. difficile* strains [33,34].

A vital aspect of CDI control is a standardized diagnosis, methods of which are summarized herein (Table 1). It is noteworthy that CDIs are frequently underdiagnosed due to persistently low awareness among doctors [35,36]. Meanwhile, an early and swift detection of hypervirulent strains is imperative, as it enables the prompt implementation of effective control measures [37]. It is essential due to the elevated mortality rate and heightened risk of infection transmission associated with strains belonging to hypervirulent ribotypes [38,39].

Reference techniques for diagnosing CDI include toxigenic strain culture and cytotoxicity tests. However, due to their labor-intensive and time-consuming nature, these techniques are commonly replaced in clinical practice by an algorithm involving immunoenzymatic tests and the detection of the glutamate dehydrogenase gene (GDH) [41,42]. Furthermore, sensitive molecular biology techniques are incorporated into the algorithm. One of the major challenges in CDI diagnosis lies in both underdiagnosis (missed detection) and overdiagnosis (interpreting asymptomatic colonization as infection). This issue may partly stem from the absence of an integrated approach in CDI detection [43,44].

The utilization of nucleic acid amplification tests (NAATs), such as real-time PCR or loop-mediated isothermal amplification, has increased in recent years [45,46]. In light of this, ribotyping stands as one of the most prevalent and stable methods for distinguishing and differentiating bacterial strains [47]. The PCR-based ribotyping technique leverages variability in copy number and size of intergenic regions between 16S and 23S rRNA genes to generate a distinct ribotype profile [48,49]. The method is based on finding differences in a highly variable *rrn* operon region, which contains genes encoding ribosomal RNA (rRNA). Each bacterial cell contains an *rrn* operon in species-dependent copy numbers, and depends on their variable amount. Given its high conservative nature, deciphering the 16S rRNA sequences has been acknowledged as the molecular “gold standard” in taxonomic classification and bacterial species identification [50,51,52]. Also, in the field of gel techniques, the use of capillary gel electrophoresis in visualizing genome fragments of 16S-23S rRNA genes has been recently applied in research for *C. difficile* ribotyping. It should be routinely used to ribotype particular strains during the diagnosis of CDI [38,53,54].

Capillary electrophoresis offers an improved separation of amplified nucleic acid fragments, leading to enhanced sensitivity of the assay compared to traditional agarose electrophoresis [52,55,56]. Therefore, it may lead to relatively fast recognition of CDI hospital outbreaks worldwide [57,58,59] and facilitate antimicrobial resistant strains’ elimination [60,61,62,63], including those derived from children [64], the environment, plants and animals [65,66,67,68,69,70,71]. As it has been previously proven, an ineffective decontamination with non-optimal time of exposure or a concentration of sporicidal disinfectants may result in an extensive *C. difficile* strain transmission [60]. This is especially possible for strains belonging to a specific hypervirulent ribotype [63]. Moreover, the use of a particular antimicrobial may improve the overall epidemiological situation on the ward [61,62].

Webribo (http://webribo.ages.at (accessed on 8 May 2024) [72] is a database which allows for the automatic analysis and comparison of capillary-sequencer-based PCR ribotyping data and efficiently simplifies laboratory PCR-ribotyping methods [57]. Moreover, an application of the mentioned database does not require own reference strains/DNA patterns of *C. difficile* strains with a particular ribotype to each investigation round, unlike the traditional ribotyping method based on PCR and agarose gel electrophoresis.

The aim of this study was to demonstrate the effectiveness of differentiation methods based on PCR ribotyping for *C. difficile* clinical strains, capillary gel electrophoresis and analysis in the Webribo database.

## 2. Results and Discussion

### 2.1. PCR Ribotyping

#### 2.1.1. Capillary Gel Electrophoresis and Analysis in the Webribo Database

**Figure 1:**
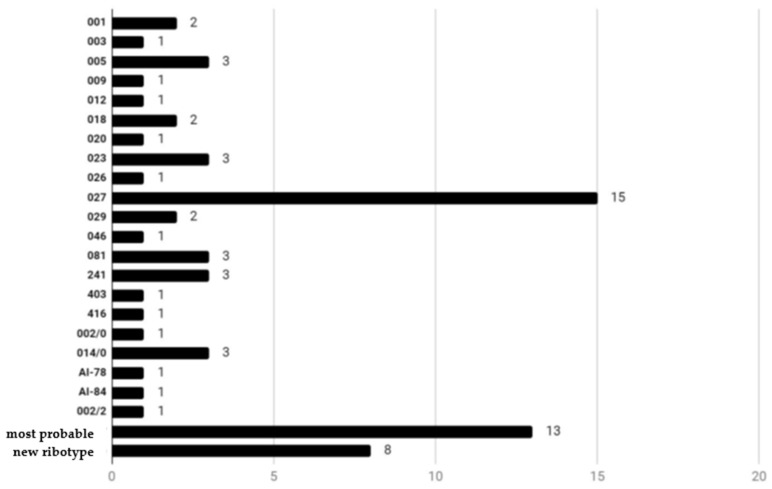
Number of the catalogued ribotypes.

According to Webribo database analysis, out of the 69 tested samples, 48 belonged to already known ribotypes, 13 represented new ribotypes and 8 indicated similarities to existing ones, with some differences, e.g., the length of fragments (all summarized in Figure 1).

The detailed results of the Webribo analysis are presented in Table 2.

Some of the ribotypes were determined by an automated algorithm to be the best fit for the selected sample. Due to the large distance, it is very likely that some of the samples established a new ribotype, and the match found by the algorithm was of no consequence.

#### 2.1.2. Toxinogenic Genes’ Presence among the Investigated Strains—Ribotype and Genotype Correlation Analysis

Upon analyzing the data from this study using the Webribo database, it was confirmed that 027 is the most frequently appearing ribotype. Out of five existing ribotypes, genotype matching could not be achieved. For samples 3, 4, 5 and 6, the contents of *tcdA*, *tcdB* and *cdtA/cdtB* were excluded. For sample 17 at the same time, ribotype 009 in the Webribo system did not find toxin content. Compared to the ribotypes defined as the most likely to appear, only two out of eight samples matched the Webribo database (Table 3).

Samples 33 and 14 were determined to be the probable ribotype 014/0, but only the second coincides with the genotypes contained in the system (Table 4).

### 2.2. Discussion 

The pathogenicity of the *C. difficile* is due to their production of cytotoxic proteins. All of them which contain tcdA, tcdB and cdtA/cdtB can cause diarrhea and colitis, and the intensity of the manifested course of the disease depends on the amount of secreted toxins. Many studies focus on the resistance of the given strains to antibiotics, and more information is still missing for ribotypes than 027.

Rates and severity of *C. difficile* infections in American hospitals, Northern Europe and Europe have been increasing steadily since 2000 [73]. They correlate with an epidemic spread of the strain, which is characterized by a higher production of toxins than their non-toxigenic counterparts and resistance to several antibiotics. The strain that appears most often in studies from around the world is BI/NAP1/027. The mere fact of such a rapid geographical spread producing severe cases of CDI disease with fatal outcomes has been the subject of many studies.

It is noteworthy that the distribution of virulent ribotypes remains closely associated with geographical location. Snydman et al. [74] observed a change in the prevalence dynamics of the 027 ribotype in the United States. The authors highlight that this shift could be attributed to several factors, including transmission patterns, the efficacy of cleaning and infection prevention measures, and the usage of different antimicrobial agents. A study conducted by Tóth et al. [57] in Hungary revealed regional variations in the prevalence of distinct ribotypes. Discrepancies were noted between the central and southeastern regions of Hungary, with the occurrence of BI/NAP1/027 isolates accounting for 45.8% in the central region and 20.8% in the southeastern region. Conversely, the second most prevalent ribotype, 036 (19.8%), was more commonly found among isolates from the southeastern region compared to the central region, with percentages of 29.1% and 10.4% noted, respectively.

In this context, the natural ecological habitats of these bacteria, including wildlife and farm animals, may serve as significant reservoirs. A study conducted by Zhang et al. [33] underscored the importance of *C. difficile* transmission monitoring between animals intended for meat consumption and humans. The authors emphasize the necessity of future studies investigating the genetic relatedness between *C. difficile* strains found in animals and those affecting humans. Such research is crucial for enhancing our understanding of their involvement in the transmission of this pathogen. Mengoli et al. [75] also highlight a significant concern in CDI therapy, which is the absence of data regarding non-clinical transmission routes, such as through food chain or environmental sources. Alongside methodological standardization, future research endeavors should prioritize improved sampling techniques and the inclusion of diverse geographic regions. This approach is essential for gaining a more precise understanding of the dynamics of *C. difficile* transmission and developing effective strategies to disrupt it.

O’Connor et al. [76], in their study on the population of *C. difficile* strains in the USA and Canada, showed that most cases with high fatality rates were caused by the presence of the hypervirulent strain BI/NAP1/027. It is present in all of the provinces in Canada, and in at least forty US states. The studies compared genomic characteristics of ribotypes 027 and 630. The results show the majority of isolates belonging to a single phylogenetic group that are distinct from other strains. Analysis of *tcdB* sequence showed a 2.9 kb region that is approximately 87–92% identical to the region of the strain 630 and encodes a unique amino acid sequence. These amino acids contain the binding domain of the toxin B receptors, which may facilitate binding to various receptors, and thus contribute to increased virulence in strains BI/NAP1/027.

Different approaches are applied for molecular studies involving *C. difficile* strains. In a study by Schneeberg [77], a test based on a DNA microarray was presented. Probes were designed to query a modularly engineered intergenic spacer region called the ISR. It is also a matrix to the conventional PCR ribotyping method using capillary electrophoresis. Forty-eight co-ribotypes were examined, resulting in the disclosure of 27 matrix profiles. The most often obtained pathogenic human ribotypes were as follows: 014/020, 001, 027 and 078/126. The study also confirmed the genetic relationship between ribotypes. PCR ribotyping was performed under the same conditions and by using the same primers as the study performed in this work.

The results of the sequential PCR ribotyping may slightly differ from the predicted ribotyping results, based on the applied strains’ genome sequences and applied methodology. Full genomes belonging to ribotype 027, e.g., CD196, differ from each other by at least one ISR length, which indicates partially spurious DNA sequencing results or an insufficient attribution of the PCR ribotype. Ribotypes 078 and 126 as well as 014 and 020 in traditional ribotyping are difficult to distinguish. With sequential PCR ribotyping, they can be made more distinctly distinguished which works better for epidemiological studies.

Of note, in 2009, 76 different isolates were sent from patients with severe CDI for testing to the national reference laboratory *C. difficile* in Germany [78] (Institute for Medical Microbiology, University of Mainz or Robert Koch Institute). PCR ribotyping was performed according to the Bidet protocol, and electrophoresis was also applied on 1.5% agarose gels in 1× TBE. Twenty-four isolates identified as 027, eight as 001 as well as 017, 042, 003, 066, 078 and 081 and a new one was named RKI-034.

In 2005, the European research group on *C. difficile* conducted studies in 38 hospitals from 14 countries over a two-month period. Out of 322, 31 toxigenic isolates were detected, 20 belonged to type 027. In January 2006 in Belgium, 896 bacterial isolates were analyzed in the reference laboratory with the ribotype 027 appearing in 158 or about 18% of them. Other common ones included ribotypes 078 (6.3%) and 031 (5.6%) [79].

In the research conducted by Lyytikäinen et al., the incidence of CDI was 1.7 per 1000 admissions; 68% of the cases appeared more than two days after admission to the hospital. The first case of the disease in Finland associated with *C. difficile*, for which 027 was responsible, was detected in 2007. Clinical laboratories in Helsinki and Turku, out of 268 isolates, isolated 131 belonging to the type 027, which is as much as 49%. The remaining ones were divided into more than 30 different ribotypes [80].

In Polish studies conducted by Pituch [81], the technique used for *C. difficile* strain typing was PCR ribotyping with a comparison of the intergenic region of 16S rRNA and 23S rRNA within strains negative for toxin A but positive for toxin B synthesis. This region is subject to frequent mutations. Materials with a deletion of the toxin A gene have been shown to exhibit genetic similarity between strains. Nine of them were used in the mentioned study on reference *C. difficile* strains isolated from patients with antibiotic-associated diarrhea, which produced toxin B but did not produce toxin A. PCR was performed using primers specific for the 3′ 16S rRNA gene end and the 5′ end of 23S rRNA. Then, pulse electrophoresis and DNA preparation in agarose blocks were performed; restriction enzyme digestion and agarose gel electrophoresis were also conducted. The results show that all the strains belonged to one ribotype, which is inconsistent with the results of our study. The author of the work himself emphasizes the difficulties in typing strains using pulsed field gel electrophoresis. In some strains, no ribotypes were obtained, which could have been caused by DNA degradation due to powerful bacterial nucleases, while the use of PCR ribotyping and capillary gel electrophoresis achieved ribotype profiles in a much shorter time and without having to perform a large number of laboratory steps. At the same time, the results were clearer and more accurate with the possibility of multiple samples testing at the same time. Another difference was found in the acquisition samples by the author from various medical facilities in a large interval of time. Samples used for this research came from one hospital only.

Research by Indra [55] in capillary gel electrophoresis shows 11 fragments per isolate. Each had a minimum size of 233 bp and a maximum size of 680 bp. Among the isolates of one ribotype, size differences in conventional electrophoresis agarose gel were as high as 27 bp for some fragments. Capillary electrophoresis results for individual peaks within one ribotype did not exceed deviations of more than one bp. For 146 tested strains, 47 obtained Austrian ribotype patterns. The production of enterotoxin A and cytotoxin B in vitro was additionally detected. The exception was isolate 017 and one AI-51 in which a negative result was obtained for toxin A. Binary toxin genes were found in 10 out of 141 isolates, which is about 7%. Compared to the research in this work, enterotoxin A and cytotoxin B were also detected in almost all the tested strains. Binary toxin gene was found in almost 50% of the isolates.

A database that collects information on *C. difficile* ribotypes and their genotypes in its analysis compares not only the lengths of the reads, but also the reaction conditions. All existing ribotypes were determined using an internal primer LIZ 1200 (for details, see Supplementary Materials). New ribotypes and possibly matching existing ones were frequently analyzed using different equipment, as well as standard and different reaction conditions. Thanks to appropriately selected algorithms and a small number of available internal standards, all the differences can be read worldwide in a few minutes to compare results from other laboratories.

Based on data from the Webribo database, the number of different ribotypes was determined in individual countries. The most different *C. difficile* ribotypes have been described in Austria, Poland, Czech Republic, and Great Britain. Many factors can affect the spread of *C. difficile*, i.e., hospital overcrowding and staff shortages, an aging population of hospital patients and the intensification of comorbidities, a high level use of antibiotics (in particular fluoroquinolones), worldwide travels (which are becoming increasingly popular) as well as hospital transfers [75].

The management of antibiotic dosing plays a crucial role in controlling and influencing *C. difficile* infections [82]. This was confirmed by a study conducted by Vaverková et al. [63], which investigated the effects of restricting the use of fluoroquinolone antibiotics on *C. difficile* infections. The study, conducted at the University Hospital in Hradec Králové, demonstrated that an effective antibiotic management strategy led to a 23.2% decrease in the incidence of CDI and a reduction in the prevalence of hypervirulent ribotypes 001 and 176. Additionally, research conducted in the United States has shown that the decreased usage of fluoroquinolones within a large healthcare system results in an uptick in the utilization of broad-spectrum cephalosporins. This shift contributes to a decline in CDI cases and enhances resistance patterns [61].

To be well acquainted with the dissemination routes, variability, and frequency occurrence of individual *C. difficile* strains, including relevant clinical strains, it is necessary to collect much more data from different countries and geographical regions. The ribotyping technique used in this work is a relatively cheap, quick and effective method that could be used in monitoring the spread of individual strains of this species. In summary, expanding data collection, efforts across diverse geographic locations and utilizing efficient techniques such as ribotyping are essential steps toward a better understanding of the transmission patterns and characteristics of *C. difficile* strains. By leveraging these methods, we can enhance surveillance capabilities and implement targeted interventions to mitigate the impact of this pathogen on public health.

## 3. Conclusions

1. PCR ribotyping is an effective method for the differentiation of *C. difficile* isolates and can be recognized as a very useful tool in epidemiological studies.
2. Due to automatic ribotype assignment in the Webribo database, the use of capillary gel electrophoresis significantly shortens the time for *C. difficile* strains ribotyping data analysis.
3. The results obtained are highly reproducible, independent of the used reagents’ batches or brands, which make it possible to compare data from different laboratories.
4. The most common *C. difficile* ribotype in the studied hospital-derived population is 027.
5. Of the remaining strains detected with toxigenic potential, all contain cytotoxin A, enterotoxin B genes or the gene encoding a binary toxin.
6. The Webribo database is a useful and an accessible database for a quick analysis of *C. difficile* ribotypes.

## 4. Materials and Methods

### 4.1. Bacterial Strains

The material for the study consisted of 69 samples of DNA obtained from *C. difficile* strains, with marked genotypes. All the strains were cultured from stool samples derived from the patients of University Hospital No. 1 in Bydgoszcz, Poland. The strains were further collected, and cultures were prepared with the DNA isolated in the Department of Microbiology of Ludwik Rydygier Collegium Medicum in Bydgoszcz, Nicolaus Copernicus University in Toruń.

### 4.2. Bacterial DNA Isolation

The DNA extraction was carried out using a Genomic Mini kit (A&A Biotechnology, Gdynia, Poland) in accordance with the manufacturer’s instructions, with the addition of lysozyme (Sigma, St. Louis, MO, USA) to improve DNA isolation efficiency from the strains. Prior to their use for the study, all the DNA samples were kept in 4 °C.

### 4.3. DNA Amplification and Amplicons’ Denaturation

Primers FAM-16S and 23S were applied for PCR-based strain differentiation. Their sequences were as follows: 16S 5′-GTGCGGCTGGATCACCTCCT-3 and 23S 5′-CCCTGCACCCTTAATAACTTGACC-3′, according to the ECDC procedure available on the website: https://www.ecdc.europa.eu/en/publications-data/laboratory-procedures-diagnosis-and-typing-human-clostridium-difficile-infection (accessed on 10 May 2024) [83].

The amplification reaction was carried out in a volume of 50 µL. The reaction mixture was prepared in a 1.5 mL Eppendorf tube placed on ice. In order to reduce the risk of contamination, the entire preparation process took place under a laminar flow chamber, in sterile conditions. To each tube containing 48 µL of the mixture, 2 µL of the isolated DNA sample was added.

The PCR reaction was carried out in on Applied Biosystems 9600 thermal cycler according to the following program: one cycle of pre-denaturation 95 °C for 5 min and 30 cycles of the following:

a. Denaturation 94 °C for 1 min;
b. Primer annealing 60 °C for 1 min;
c. Primer elongation at 72 °C for 1 min;
d. Final elongation at 72 °C for 30 min.

The procedure of fragment denaturation was as follows:

a. Reagent mix was prepared for fragment denaturation.
b. Standard size (LZ 1200) in a volume of 5 μL was dissolved in Hi-Di formamide in a volume of 100 μL.
c. The mix prepared in this way was applied to a 96-well plate in a volume of 10 μL.
d. One microleter of PCR product was added to each mixed well.
e. The whole volume was centrifuged briefly.
f. Denaturation in a thermal cycler for 2 min at 95 °C.
g. The well was placed on ice and held for about 10 min.
h. The whole solution was centrifuged briefly.

### 4.4. Fragment Analysis by Capillary Gel Electrophoresis

Electrophoresis was performed on the Applied Biosystem 3130xl Genetic Analyzer. Gel electrophoresis was performed under the following conditions:

- Laser power: 15 mW;
- A 36 cm capillary type;
- working temperature: 60 °C;
- POP7 polymer;
- Standard-sized GeneScan™ 1200 LIZ;
- A 1× EDTA buffer.

The obtained results were analyzed using the GeneMapper 3.2 software, which allows for determining the size of the obtained DNA fragments based on the internal standard LIZ 1200 (see Supplementary Materials). The obtained fragment size and peak height measurements were exported to a text file, which was then processed in MS Excel 2016 to prepare a batch file for ribotype similarity analysis in the Webribo database.

### 4.5. Assignment of Ribotypes Using the Webribo Database

A text file containing the numbers of the tested samples and the sizes of DNA fragments and peak heights, prepared in accordance with the recommendations of the database creators, was entered into the Webribo database.

The obtained ribotyping results were downloaded from the database in a text file format and imported to MS Excel for statistical analysis.

### 4.6. Evaluation of the Toxinogenic Potential of the Strain

Toxinogenic potential (*cdtA*/*cdtB*—binary toxin genes; *tcdA*—toxin A gene; *tcdB*—toxin B gene) of the investigated strains was evaluated according to ECDC procedure available on the following website: https://www.ecdc.europa.eu/en/publications-data/laboratory-procedures-diagnosis-and-typing-human-clostridium-difficile-infection (accessed on 10 May 2024).

## Figures and Tables

**Table 1:** Summary of methods for detecting *C. difficile*, along with the advantages and disadvantages of each method [40].

Test	Methodology	Advantages	Disadvantages
Stool culture	detection of bacteria	high sensitivity	necessity of a culture under anaerobic conditions, time length for the result, moderate specificity (lack of differentiation of toxigenic strains)
PCR test	detection of bacterial DNA (e.g., *GDH*, toxin genes)	high sensitivity and specificity	lack of specificity against toxin-producing strains if only *GDH* is detected
Cytotoxicity neutralization test in the cell culture	toxin detection	“gold standard” in the diagnosis of *C. difficile* intoxication; high sensitivity and specificity	labor-intensive, time-consuming, the need for constant cell culture
Immunoenzymatic tests for toxin antigens	toxin detection	speed and simplicity of testing; low-cost method	low sensitivity
PCR test (detection of toxin genes directly from stool sample or cultured strain)	toxin detection	high sensitivity and specificity, can be used as a single diagnostic method	requires appropriate equipment
GDH and toxin A/B detection test	detection of bacteria and toxin detection	fast method (2–6 h); easy to perform	low sensitivity compared to PCR and cytotoxicity tests

**Table 2:** The detailed results obtained in the research using the Webribo database.

Strain Number	Ribotype Detected	Commentary/Interpretation
10	241	Existing ribotype
101	027	Existing ribotype
102	003	Existing ribotype
103	027	Existing ribotype
106	PR09247	New ribotype
111	027	Existing ribotype
115	027	Existing ribotype
12	029	Existing ribotype
13	027	Existing ribotype
14	PR24695	Most probable: 014/0 ribotype
15	027	Existing ribotype
16	PR24696	New ribotype
17	009	Existing ribotype
18	027	Existing ribotype
19	018	Existing ribotype
21	027	Existing ribotype
22	PR2468	Most probable: 076 ribotype
23	PR24697	New ribotype
24	027	Existing ribotype
25	027	Existing ribotype
26	AI-84	Existing ribotype
27	PR24698	Most probable: AI-61 ribotype
3	081	Existing ribotype
30	241	Existing ribotype
32	005	Existing ribotype
33	PR24695	Most probable: 014/0 ribotype
34	014/0	Existing ribotype
36	PR24694	Most probable: 006/1 ribotype
37	005	Existing ribotype
38	241	Existing ribotype
39	023	Existing ribotype
4	002/2	Existing ribotype
40	020	Existing ribotype
41	416	Existing ribotype
42	027	Existing ribotype
43	029	Existing ribotype
44	403	Existing ribotype
45	081	Existing ribotype
46	027	Existing ribotype
47	PR24683	New ribotype
48	PR24699	New ribotype
49	PR24700	Most probable: 095 ribotype
5	005	Existing ribotype
50	046	Existing ribotype
51	PR24701	New ribotype
52	PR24702	New ribotype
53	014/0	Existing ribotype
54	PR01729	New ribotype
55	PR01744	Most probable: 031 ribotype
56	027	Existing ribotype
57	001	Existing ribotype
58	PR24684	New ribotype
59	012	Existing ribotype
6	018	Existing ribotype
60	AI-78	Existing ribotype
7	PR24685	New ribotype
70	PR01729	New ribotype
71	PR24686	New ribotype
72	014/0	Existing ribotype
73	PR24687	New ribotype
74	001	Existing ribotype
77	027	Existing ribotype
78	027	Existing ribotype
79	PR24690	Most probable: 578 ribotype
8	002/0	Existing ribotype
9	026	Existing ribotype
91	023	Existing ribotype
92	081	Existing ribotype
93	023	Existing ribotype

**Table 3:** Summary of the existing ribotypes obtained in the research using the Webribo database with the observed genotypes.

Ribotype Detected	Strain Number	*tcdA*	*tcdB*	*cdtA/cdtB*	Ribotype Detected	Strain Number	*tcdA*	*tcdB*	*cdtA/cdtB*
027	56	pos.	pos.	pos.	020	40	pos.	pos.	neg.
	46	pos.	pos.	pos.	241	10	pos.	pos.	neg.
	103	pos.	pos.	pos.		38	pos.	pos.	neg.
	115	pos.	pos.	pos.		30	pos.	pos.	neg.
	111	pos.	pos.	pos.	0/12	59	pos.	pos.	neg.
	13	pos.	pos.	pos.	002/2	4	pos.	pos.	neg.
	18	pos.	pos.	pos.	0/18	19	pos.	pos.	neg.
	21	pos.	pos.	pos.		6	pos.	pos.	neg.
	15	pos.	pos.	pos.	005	5	pos.	pos.	neg.
	24	pos.	pos.	pos.		32	pos.	pos.	neg.
	25	pos.	pos.	pos.		37	pos.	pos.	neg.
	42	pos.	pos.	pos.	023	93	pos.	pos.	pos.
	101	pos.	pos.	pos.		91	pos.	pos.	pos.
	77	pos.	pos.	pos.		39	pos.	pos.	pos.
	78	pos.	pos.	pos.	081	3	pos.	pos.	neg.
014/0	34	pos.	pos.	neg.		45	pos.	pos.	neg.
	53	pos.	pos.	neg.		92	pos.	pos.	neg.
	72	pos.	pos.	neg.	029	43	pos.	pos.	neg.
001	74	pos.	pos.	pos.		12	pos.	pos.	neg.
	57	pos.	pos.	pos.	003	102	pos.	pos.	neg.
009	17	pos.	pos.	neg.					

*cdtA/cdtB*—binary toxin genes; neg.—gene absence; pos.—gene presence; *tcdA*—toxin A gene; *tcdB*—toxin B gene.

**Table 4:** Summary of the most probable existing ribotypes obtained in the research using the Webribo database with respect to the observed genotypes.

Ribotype Detected	The Most Probable Ribotype	Strain Number	*tcdA*	*tcdB*	*cdtA/cdtB*
PR24695	014/0	14	pos.	pos.	pos.
PR24689	076	22	pos.	pos.	pos.
PR24698	AI-61	27	neg.	neg.	neg.
PR24694	006/1	36	pos.	pos.	neg.
PR24700	095	49	pos.	pos.	neg.
PR01744	031	55	pos.	pos.	neg.
PR24690	578	79	pos.	pos.	pos.
PR24695	014/0	33	pos.	pos.	neg.

*cdtA/cdtB*—binary toxin genes; neg.—gene absence; pos.—gene presence; *tcdA*—toxin A gene; *tcdB*—toxin B gene.

## Data Availability

The data presented in this study are available on request from the corresponding author.

## Supplementary Materials

The following supporting information can be downloaded at: https://www.mdpi.com/article/10.3390/gels10050343/s1, Figure S1: An example of a perfect ribotype match (ribotype 027); Figure S2: An example of a ribotype identified as the “most probable ribotype” (014/0); Figure S3: An example of a new ribotype.
