# Supplementary figures and images for: Usefulness of Capillary Gel Electrophoresis-Based PCR for Detection of Clostridioides difficile Strains with Hypervirulent Ribotypes
Source: Gels. 2024 May 17;10(5):343. doi: 10.3390/gels10050343 (PMC11121280; doi:10.3390/gels10050343)

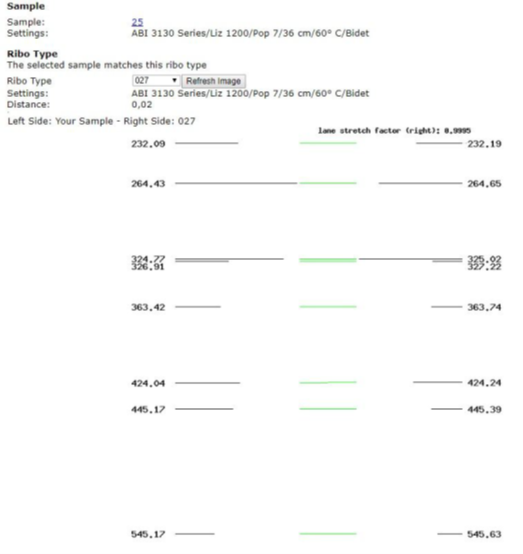

Supplement: Supplementary file 1 [file gels-10-00343-s001.zip › Figure S1.png]

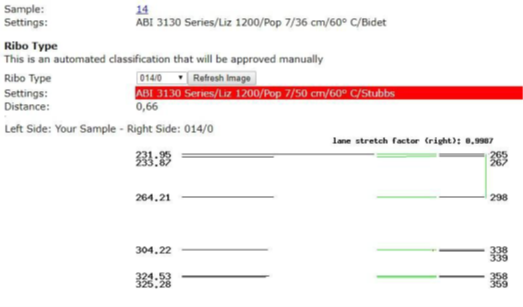

Supplement: Supplementary file 1 [file gels-10-00343-s001.zip › Figure S2.png]

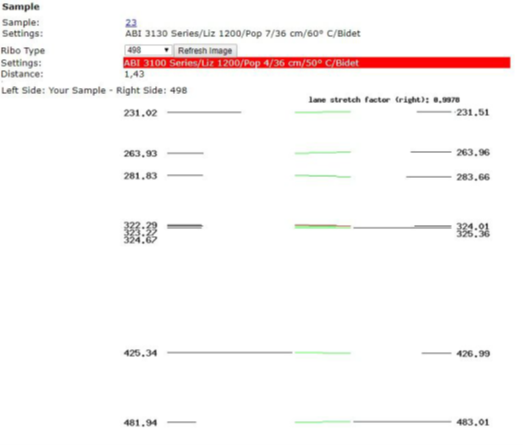

Supplement: Supplementary file 1 [file gels-10-00343-s001.zip › Figure S3.png]
